# Myxoid liposarcoma in a 91-year-old patient

**DOI:** 10.1186/1755-8166-6-50

**Published:** 2013-11-19

**Authors:** Brandon S Sheffield, Torsten O Nielsen

**Affiliations:** 1 Department of pathology and laboratory medicine, University of British Columbia, 899 West 12th Avenue, Vancouver, British Columbia V5Z 1M9, Canada

**Keywords:** Liposarcoma, Myxoid liposarcoma, Round cell liposarcoma, Advanced age, DDIT3, FUS, FISH

## Abstract

**Background:**

Myxoid liposarcoma is a mesenchymal malignancy most commonly presenting in young adults. This tumor is known for its characteristic chromosomal rearrangement at the *DDIT3* locus.

**Results:**

We report a case of myxoid liposarcoma in a 91-year-old, the oldest known patient with this disease-entity. FISH analysis of the *DDIT3* and *FUS* loci demonstrate the pathognomonic chromosomal alteration in the setting of predominantly round cell histology on biopsy, confirmed by RT-PCR.

**Conclusion:**

Myxoid liposarcoma affects mostly young adults but can be seen in the elderly population. Molecular and cytogenetic assays are helpful auxiliaries to histology in the setting of unusual histology and clinical presentation.

## Background

Myxoid liposarcoma, the second-most common subtype of liposarcoma, accounts for 10% of soft tissue sarcomas [1]. Myxoid liposarcoma tends to occur on extremities, with two thirds of cases originating in the thigh [2]. This disease affects a younger demographic than most cancers with peak incidence between ages 30–50 [1], and is the most common subtype of liposarcoma in the pediatric age group [3].

Typical myxoid liposarcoma histology shows nonpleomorphic ovoid mesenchymal cells and rare lipoblasts amidst a prominent myxoid stroma, with an intricately plexiform vasculature. Round-cell liposarcoma, a high-grade form of myxoid liposarcoma, lacks myxoid matrix leaving closely packed, round mesenchymal cells. Regions of myxoid and round-cell liposarcoma morphologies often coexist (MLS/RCLS).

Both morphologies share identical cytogenetic aberrations and are thus considered as a single pathologic entity [1]. A reciprocal translocation *t(12;16)* can be identified in the majority of cases [4] creating an in-frame fusion of *FUS* and *DDIT3* that is specific for MLS/RCLS [5]. A small proportion of cases harbor an alternate *t(12;22) EWS-DDIT3* rearrangement [6]. Demonstration of disruption at the *DDIT3* locus is an effective diagnostic tool for MLS/RCLS [7], with break-apart fluorescent *in situ* hybridization (FISH) probes commercially available.

The most powerful prognostic marker in MLS/RCLS is the presence of a round cell component comprising greater than 5% of the tumor. These high grade round cell liposarcaromas have been shown to behave more aggressively with decreased overall survival and metastasis free survival [8].

Compared with other soft tissue sarcomas, MLS/RCLS is particularly sensitive to radiation therapy [9]. Accordingly, neoadjuvant radiation has been recommended in the treatment of myxoid liposarcoma [10]. This finding places increased significance on accurate diagnosis prior to surgical resection.

The following report depicts a case of MLS/RCLS in a rare elderly age demographic while highlighting the salient diagnostic features of this disease, and the value of molecular testing in the context of unusual clinical features and histology.

## Case presentation

A 91-year-old male presented to a local emergency department complaining of a large mass in the medial thigh that had grown in size over the preceding several weeks. The mass was otherwise asymptomatic.

The patient’s past medical history included atrial fibrillation and hypertension, prostate cancer treated by resection with local recurrence treated by radiotherapy 20 years previously, laryngeal cancer treated by radiotherapy 14 years previously, and finally, separate histories of both colon and anal cancer treated with an abdominal-perineal resection 6 years previously. The patient reported a family history of ovarian cancer and multiple prostate cancers.

**Figure 1:**
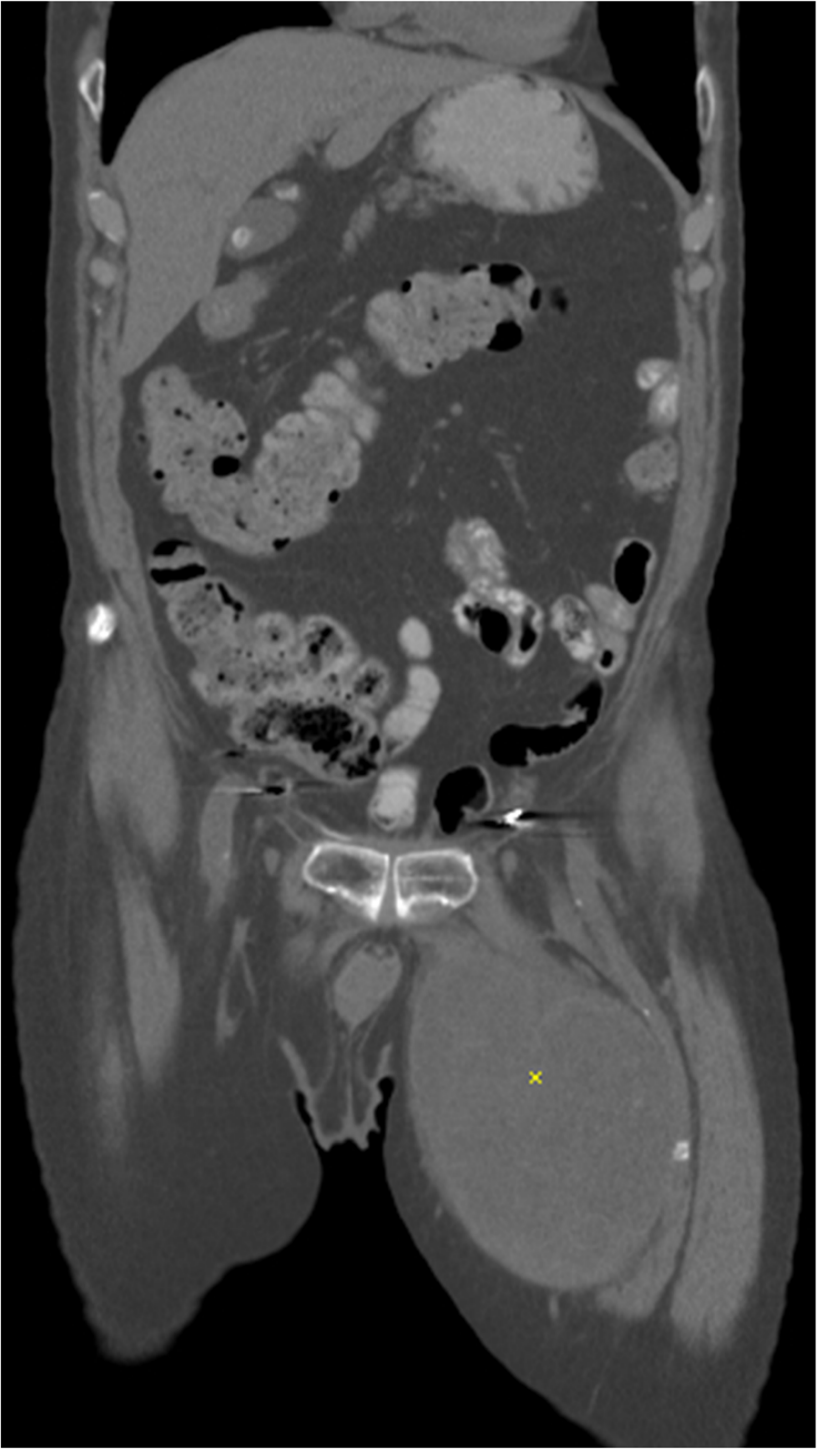
Abdomino-pelvic CT scan showing tumor in the left medial thigh (*).

CT scan of the pelvis demonstrated a 19 cm lobulated mass with complex septations and central necrosis in the adductor compartment of the left thigh (Figure 1).

**Figure 2:**
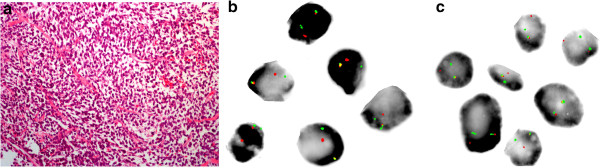
**Core needle biopsy of mass. (a)** 20× H&E section showing hypercellularity, and lacking any differentiated morphology. **(b)** and **(c)** Fluorescent photomicrographs showing biopsied tumor cells hybridized to commercial break-apart probes specific to the *DDIT3***(b)** and *FUS***(c)** loci. Probes flank targeted gene sequence showing yellow signal when bound in proximity (intact locus) and showing individual red and or green signals when bound in isolation (rearranged locus). *EWSR1* (not shown) was conversely intact with two paired (yellow) signals per nucleus.

Core needle biopsy of the mass under ultrasound guidance showed high-grade spindled, ovoid and round cell morphology. Although a small amount of myxoid matrix and complex vasculature were noted, no unequivocal lipoblasts were evident and the tumor was considered to lack differentiation towards any specific lineage (Figure 2a). Immunohistochemical workup was nonspecific, with the tumor showing positive staining for S-100 and weak staining for CD34 and BCL-2. No immunoreactivity was present for melan-A, pankeratin, desmin, smooth-muscle actin, TLE, CD20, CD43, or CD99. Mitotic figures were numerous and the Ki67 index was high. With no specific diagnosis evident after this workup, in the context of blue cell histology for a large soft tissue mass, FISH analysis was performed for *DDIT3*, *FUS¸* and *EWSR1*. Break-apart probes for *DDIT3* and *FUS* demonstrated chromosomal rearrangements at both of these loci (Figures 2b, c), while *EWSR1* was intact. RT-PCR confirmed expression of a type 1 *FUS*exon7-*DDIT3*exon2 fusion transcript. The RT-PCR was performed according to previously established protocols [11], using oligonucleotide primers to exon 6 of FUS (5′-gaacccagaggtcgtggag-3′) and exon 2 of DDIT3 (5′-tgctttcaggtgtggtgatg-3′).

**Figure 3:**
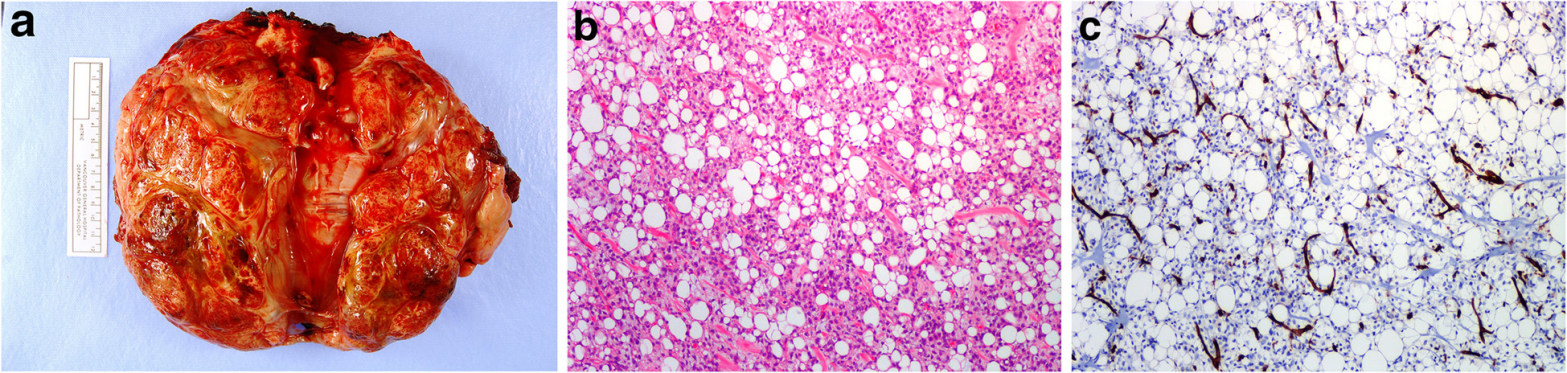
**Excision of mass.** Gross photo showing 19 cm yellow, hemorrhagic mass **(a)**, featuring regions with typical myxoid liposarcoma, 10× H&E **(b)**, and typical chicken-wire vasculature highlighted by CD31 immunohistochemical stain **(c)**.

Based on the pathognomonic molecular findings, a diagnosis of MLS/RCLS was made. The patient was discussed at multidisciplinary tumor board, and offered neoadjuvant radiation treatment followed by surgical excision. Both radiation and surgery were well-tolerated. Final pathology showed a 19 cm lobulated heterogenous yellow and hemorrhagic mass (Figure 3a). Microscopic sections showed a mixture of high-grade round cell areas similar to that seen at biopsy, as well as regions showing a more typical myxoid liposarcoma histology that had not been evident on the core biopsy (Figures 3b, c), providing morphological confirmation of the diagnosis.

A search of the medical literature was performed for reports of myxoid and/or round cell liposarcoma in the elderly. Moreau *et al.*[10] recently published a series of 418 MLS/RCLS patients, the oldest being 85 years old at presentation. To our knowledge, the current case represents the oldest reported age for a patient presenting with primary myxoid or round cell liposarcoma.

## Discussion

Several facets of myxoid liposarcoma diagnosis and treatment are highlighted in this case. The diagnosis of myxoid liposarcoma in an elderly patient is uncommon, but does occur. Thus an advanced age should not exclude myxoid liposarcoma when other clinical, radiologic, histologic, or molecular findings support this diagnosis.

Myxoid liposarcoma can show a wide variation of histologic patterns, at times within the same tumor [12]. This can lead to difficult diagnoses, especially on small biopsy specimens. This case highlights the value of molecular diagnosis as an adjunct to histology. FISH is available in specialty centers, and is available to most surgical pathology labs as it can be performed on standard formalin-fixed, paraffin-embedded tissue sections (shipped to central labs if needed). In the current era of diagnostics, molecular confirmation of sarcomas is quickly becoming a standard of care for several types of sarcomas bearing pathognomonic genetic events. The availability of molecular diagnostic tools may, in part, contribute to the overall improved outcomes observed in sarcoma patients treated at higher volume institutions [13].

The accurate preoperative diagnosis of myxoid liposarcoma prioritized neoadjuvant radiotherapy as the next step in management. This treatment modality has particular benefit in myxoid liposarcoma [9]. Accurate diagnosis on biopsy in elderly patients may have additional value if poor baseline functioning precludes the patient as a surgical candidate, as palliative radiation may be particularly effective after a diagnosis of myxoid liposarcoma.

## Conclusion

Myxoid/round cell liposarcoma is a relatively common sarcoma among younger adults presenting with a large, deep-seated malignant primary soft tissue mass. However, this tumor can affect older individuals. Molecular cytogenetic studies can greatly assist diagnosis in cases with unusual clinical and histologic features.

## Consent

Written informed consent was obtained from the patient for publication of this case report and any accompanying images. A copy of the written consent is available for review by the Editor-in-Chief of this journal.

## Competing interests

The authors declare that they have no competing interest.

## Authors’ contributions

BSS Assisted in interpreting the diagnostic biopsy, compiled the case report, and wrote the manuscript. TON Issued pathological diagnosis of the biopsy and resection specimens, performed cytogenetic and molecular workup, edited the manuscript, and supervised the project. Both authors read and approved the final manuscript.
